# The Book World of Medicine and Science

**Published:** 1896-10-17

**Authors:** 


### THE BOOK WORLD OF MEDICINE AND SCIENCE.

The Surgery of the Chest. By Stephen Paget, M.A., F.R.C.S., Surgeon to the West London Hospital, and to the Metropolitan Hospital, Bristol. (John Wright and Co., 1896. Demy 8vo., 480 pp. 12 plates. 10s. 6d. net.)

Mr. Paget has produced an excellent and representative work, stating the case for the surgery of the chest, as it now stands, clearly and fairly. He argues without bias, and largely quotes from the evidence of others, so that the book is a most interesting and complete exposition written in charming literary style. The material is throughout illustrated by typical cases, to which we would gladly have seen the references appended. Two sections deal with injuries and diseases respectively. The first chapter is short and incomplete, on landmarks and congenital malformations. Next the importance of concussion of the chest is made clear, and a valuable article on fractures and dislocation of ribs, cartilages, and sternum appears. The rest of the first section of the book is taken up with complications affecting the lung, heart, &c. Surgical emphysema and pneumothorax are fully described, and early puncture for the latter recommended. Pneumothorax, like emphysema, is really expiratory in origin, due to forcible separation of the pleural surfaces, as shown by Dr. West. The expiratory view is also applied to hernia of the lung. The chapter on wounds of the intrathorcaic vessels is followed by that of wounds of the lung, concerning which he quotes Rose, "Certainly the worst thing of all for the patient is throrough examination," and lays stress on the fact that absence of hæmoptysis does not exclude lung injury. There is an interesting description of wounds of the heart, with a table of eight cases with five recoveries, and the therapeutic value of prolonged rest is insisted on. Then follow injuries and herniæ connected with the diaphragm. In Part 2 the risk of sudden œdema of the lung after rapid drainage of pleural effusion is pointed out. Eighty pages are rightly devoted to empyema. "The wheel has come full circle; the discoveries of Lister have brought us back to the free incision practised by Hippocrates." There is a useful chapter on foreign bodies contained in the air passages. Pneumonectomy for phthisis is denounced. Puncture of the heart in chloroform syncope, so successful in animals, has proved useless in man. Under pericardial effusion Dr. Ewart's practical classification of the signs is quoted in full. The work is completed with the considerations of new growths, hydatid, actinomycosis, aspergillus, and last, but not least, subphrenic abscess. It is a work which is a credit to the author and to British surgery, but in the next edition we shall look forward to illustrations more worthy of the text.

Angio-Neurosis: Being Studies in Diseases of the Vaso-Motor System. By W. Ramsay Smith, M.B., C.M., B.Sc. (Bristol: John Wright and Co. Pp. 78. Price 4s. net.)

This is a description of two diseases which form part of a chain of morbid states ranging from Graves's disease at the one end to Raynaud's disease at the other. The two conditions which the author deals with in the book before us he has named General Angio-neurotic Œdema, and Erythema-urticaria.

In the first of these conditions the attack comes on suddenly, and is attended with high temperature, hyperæmia, and swelling of the skin, and is followed by desquamation, and in some cases the patients show a tendency to suffer repeatedly from similar attacks. The malady also occurs in a localised form, in which, however, there is no rise of temperature.

The second condition, erythema-urticaria, seems fairly described by its compound name. It is interesting to note that, according to the author, it is apt to take on an epidemic form among those who are subject to it, occurring among many of them about the same time.

After giving a careful description of the maladies to which he wishes to direct attention, the author enters into a general discussion of the *rôle* played by the various predisposing and exciting factors in the production of angioneurosis. Peripheral irritation and heat, emotion, toxic, and climatic conditions are all considered in this relation, as also is the influence of heredity.

The book is very well printed, and is illustrated by half-a-dozen reproductions from photographs of cases.

The Imperial Health Manual of Germany. (London; Baillière, Tindall, and Cox, King William Street, Strand.)

To the medical man who has regard to the well-being of his nation as a whole, it is a pleasure to see such a manua as that issued by the Imperial Health Department of Germany. The book is a well-considered attempt to show to every intelligent person the necessity and value of health, and the scientific foundations on which alone individual and national health can be built. The preface to the book contains figures of the most markedly interesting character. Measuring health value in terms of money, looking at it from the mere commercial standpoint, we are amazed to find how large is the total of loss to the community from sickness in any given year. For example, the numbers of persons belonging to sick clubs in Germany were, in 1891, about 6½ millions. The total amount paid by those sick clubs to their sick members in that year was £4,475,000. Assuming that the proportion of cases of sickness throughout the remaining 44 millions of people in Germany was equal to that among the members of the various clubs, we get a total of £25,000,000 as the cost of the sickness of the people during 1891. This amount, we are told, does not include loss of wages, or the diminished profits of sick employers, but only the money actually paid away in necessary sick expenses. It is clear then that sickness results in a very serious loss both to individuals and nations. It is equally clear also that if individuals and nations are at all sensible on this point, they will adopt any rational means which may be available for the avoidance of such waste and loss. Now the only effective means are twofold, first, the spread of scientific information, and secondly, the intelligent use of such knowledge by individuals and local and national authorities in daily life. The manual begins at the beginning, and the human body is depicted by means of clear diagrams and drawings, both as to its skeleton, and as to its detailed muscular, organic, and general structure. The letterpress accompanying the drawings is brief, plain, and clear. In like manner the various causes of bad health, and the manifold means of promoting good health, are all set forth with a clearness and precision which leave nothing to be desired. In all this the Imperial Health Department of Germany has rendered excellent service to the community. Naturally, in considering facts of such a kind, the reviewer compares foreign with home efforts in the same direction. So comparing, we are bound to admit that at least this particular thing is "done better in Germany," and the admission is made with sorrow. We wish we could believe and hope that within the next five years we should have a health department in this country as anxious to spread valuable and much-needed information as the Imperial Health Department of Germany.

